# 633. Scald Burns Associated with Hair Braiding: A Review of the Literature

**DOI:** 10.1093/jbcr/irag033.459

**Published:** 2026-04-06

**Authors:** Dharani Rao, Michael L Cooper

**Affiliations:** Jerome L. Finkelstein, MD, Regional Burn Center, New Windsor, NY; Staten Island University Hospital Northwell Health, Staten Island, NY

## Abstract

**Introduction:**

Although scald burns resulting from the practice of dipping hair braids into boiling water are a common injury, limited literature exists characterizing this burn mechanism. The practice is performed at the end of the hair braiding process in order to “set” the braids and prevent unraveling. Often the boiling water is accidentally spilled resulting in severe burns that affect both the hair braider and person receiving the braids. We aim to review the literature surrounding these practices to better characterize the context of the injury and identify trends across studies. To our knowledge there has been no previous review compiling literature findings describing this injury type.

**Methods:**

PubMed, Cochrane Library, EBSCO, Web of Science, and Embase were used to identify all literature relevant to the following search terms; “hair braiding AND scald burns.” Inclusion criteria were as follows; all literature relating to scald burn injury resulting from hair braiding. Both adult and pediatric populations were included, injury affecting both the braider and client were included as well. Studies describing chemical, flame or contact burns occurring during the hair braiding process, or wounds, injury or burns to the scalp were excluded from review. 13 articles were identified for initial review. 8 studies met inclusion criteria.

**Results:**

Among the 8 selected articles were 3 case reports/case series and 5 retrospective studies from chart review. Across studies females and the pediatric population were more frequently affected. Percent TBSA from this injury ranged from 3% to 5%, and the most affected region was the back and upper extremities. Although variability existed between studies in frequency of admission and pursuit of operative intervention vs conservative management, this injury type was more frequently addressed without operative intervention. Finally, the most frequently sited long-term complication was hypo-pigmentation of the wound site. Additionally, patients more frequently presented after being burned at home rather than in a hair salon. Studies also describe possible seasonal trends.

**Conclusions:**

While functional and cosmetic outcomes following scald burns from hair braiding are largely positive and appear similar across reports in the literature, this burn mechanism remains a frequent cause of injury associated with significant morbidity. The scope of this review was constrained by limited published data, further research is required to more conclusively evaluate other aspects of this injury pattern such as cost burden, long term psychological outcomes, and prevalence of protective garment use or injury prevention awareness among hair braiders.

**Applicability of Research to Practice:**

Safety measures, including preventative education targeting at-home braiding, are required to effectively reduce the number of affected patients. We hope to encourage further reporting and study of this injury mechanism as it remains highly prevalent.

**Funding for the study:**

N/A.

